# Metabolomics study identified bile acids as potential biomarkers for gastric cancer: A case control study

**DOI:** 10.3389/fendo.2022.1039786

**Published:** 2022-11-18

**Authors:** Chen Pan, Dawei Deng, Tianfu Wei, Zeming Wu, Biao Zhang, Qihang Yuan, Guogang Liang, Yanfeng Liu, Peiyuan Yin

**Affiliations:** ^1^ Department of General Surgery, First Affiliated Hospital of Dalian Medical University, Dalian, China; ^2^ Clinical Laboratory of Integrative Medicine, First Affiliated Hospital of Dalian Medical University, Dalian, China; ^3^ Department of General Surgery, The First Affiliated Hospital of University of Science and Technology of China (USTC), Division of Life Sciences and Medicine, University of Science and Technology of China, Hefei, China; ^4^ Department of Hepato-Biliary-Pancreas, Affiliated Hospital of North Sichuan Medical College, Nanchong, China; ^5^ iPhenome Biotechnology (Yun Pu Kang) Inc., Dalian, China; ^6^ Institute of Integrative Medicine, Dalian Medical University, Dalian, China

**Keywords:** gastric cancer, bile acids, metabolomics, biomarkers, LC MS

## Abstract

Gastric cancer (GC) is a common lethal malignancy worldwide. Gastroscopy is an effective screening technique for decreasing mortality. However, there are still limited useful non-invasive markers for early detection of GC. Bile acids are important molecules for the modulation of energy metabolism. With an in-depth targeted method for accurate quantitation of 80 bile acids (BAs), we aimed to find potential biomarkers for the early screening of GC. A cohort with 280 participants was enrolled, including 113 GC, 22 benign gastric lesions (BGL) and 145 healthy controls. Potential markers were identified using a random forest machine algorithm in the discovery cohort (n=180), then validated in an internal validation cohort (n=78) and a group with 22 BGL. The results represented significant alterations in the circulating BA pool between GC and the controls. BAs also exhibited significant correlations with various clinical traits. Then, we developed a diagnostic panel that comprised six BAs or ratios for GC detection. The panel showed high accuracy for the diagnosis of GC with AUC of 1 (95%CI: 1.00-1.00) and 0.98 (95%CI: 0.93-1.00) in the discovery and validation cohort, respectively. This 6-BAs panel was also able to identify early GC with AUC of 1 (95%CI: 0.999-1.00) and 0.94 (95%CI: 0.83-1.00) in the discovery and validation cohort, respectively. Meanwhile, this panel achieved a good differential diagnosis between GC and BGL and the AUC was 0.873 (95%CI: 0.812-0.934). The alternations of serum bile acids are characteristic metabolic features of GC. Bile acids could be promising biomarkers for the early diagnosis of GC.

## Introduction

Gastric cancer (GC) is a highly aggressive and fatal malignancy with high mortality and accounts for the second leading cause of cancer-related deaths worldwide (1, 2). Pathological grading of GC plays an essential role in determining patient prognosis (3, 4). Screening and early diagnosis of GC are critical in the prevention and treatment of GC. However, existing non-invasive tumor markers, such as carcinoembryonic antigen (CEA), have low GC evaluation efficiency, especially early gastric cancer (EGC) (5, 6). Endoscopy is commonly utilized in the screening and diagnosis of GC in clinical practice and has dramatically improved the disease outcome. However, gastroscopy consumes tremendous medical resources, and its cost-benefit remains debatable. Besides, because of its invasiveness, gastroscopy causes great anxiety to the subjects (7, 8). Therefore, current screening strategies only cover high-risk individuals who are older than 40 years or those with a prior history of gastropathy (9). There is an urgent need for innovative biomarkers to screen high-risk populations who require gastroscopy.

GC development has been associated with both genetic and environmental factors. In addition, metabolites are the end products of a complex interplay between intrinsic metabolism, environmental exposure and genetic predisposition (10). Occurrence of metabolic reprogramming in GC coupled with variations in the metabolites facilitates understanding of tumor biology. Previous metabolomic data showed that energy metabolism, amino acid metabolism and lipid metabolism are related to GC progression (11–13). Besides, GC with peritoneal metastasis depends on unique metabolic features (14). Thus, metabolomics, a new omics technique, provides a powerful tool for GC understanding.

On the other hand, bile reflux has been shown to be an independent risk factor for precancerous gastric lesions and GC (15). Bile acids (BAs), an important component of bile, play a significant role in regulating the digestive system and homeostasis of intestinal flora. A previous study demonstrated that BAs interact with the gut flora to influence human health (16). The metabolism of BAs was significantly disrupted in patients with Alzheimer’s disease (AD), which was associated with cognitive impairment (17). Increasing evidence has indicated that BAs were involved in the occurrence and development of gastrointestinal tumors. For instance, deoxycholic acid (DCA) was shown to induce the expression of hepatocyte inflammatory genes, whose long-term expression was strongly associated with hepatocellular carcinoma (HCC) (18). However, the relationship between BA metabolism disorders and GC remains unclear.

Previous studies have shown that metabolites in blood or urine have the potential to be GC biomarkers (14, 19). Since quantification of the metabolites is the ultimate goal of metabolomics (20), most of the studies had a relatively small sample size and lacked a validation cohort, thus low quantification power. Besides, most of the previous studies utilized non-targeted metabolomic methods which had poor data stability, repeatability and quantitative linear range, limiting the clinical transformation of the outcome. This study used an in-depth targeted method developed for accurate quantitation of the BAs to analyze 280 blood samples from GC patients, benign gastric lesions(BGL) and healthy controls. Our study aimed to dissect the pathophysiologic interaction between BA metabolism and GC to identify biomarkers for early diagnosis.

## Materials and methods

### Participants and criteria

Serum samples were collected from the GC patients, BGL patients and healthy participants (Con) at the First Affiliated Hospital of Dalian Medical University from May 2020 to October 2021. The GC patients were pathologically diagnosed by biopsy and divided into sub-groups based on the AJCC staging system, 8th edition (21). The sub-groups included the degree of differentiation, TNM stage as well as early or advanced GC. Samples in the Con group were collected from healthy participants during physical examination, and there was no obvious abnormality as assessed by gastroscopy. The age and gender ratio of the Con group were matched with the GC group. We excluded patients in the GC who had other forms of cancer, liver or renal insufficiency, severe cardiopulmonary diseases, metabolic diseases, active bleeding, and other mental or physical diseases. All 22 cases of BGL were confirmed by pathological biopsy. It mainly included chronic atrophic gastritis, adenomatous polyps, gastric ulcer and low grade intraepithelial neoplasia. On the other hand, we excluded any patients who had a history of gastrointestinal diseases, such as acute or chronic gastritis, upper gastrointestinal ulcers, upper gastrointestinal perforation, gastroesophageal reflux disease or benign tumors in the Con group. All the participants signed informed consent forms, and the study was approved by the Ethics Committee of First Affiliated Hospital of Dalian Medical University.

### Serum sample collection and pretreatment

The GC patient serum samples under fasting were collected on the first morning after admission. All the BGL and Con samples were collected simultaneously and under the same fasting conditions as the GC samples. The samples were then stored at -80°C and thawed before pretreatment. At the beginning of the pretreatment, we transferred 80 μL of serum to 1 mL 96-well plate, then added 320 μL of mixed BA isotope internal standard dissolved in acetonitrile: methanol (1:1, v:v), as shown in **Table S4**. After 3 minutes of vortexing, the solution was then centrifuged for 20 minutes. We transferred 260 μL of the supernatant to another 96-well plate and then dried the extraction by centrifugal vacuum concentration (Labconco Corporation, USA). The remaining supernatant in all samples was mixed and distributed at the same volume as those in quality control (QC) samples (22–24). Before the BA-targeted metabolomic analysis, the extraction was redissolved in 50% methanol in water.

### Metabolomic analysis

After injection of 2.5 μL of redissolved BA extraction, a total of 63 BAs (**Table S5**) were target detected by a Shimadzu UPLC (Shimadzu, Kyoto, Japan), coupled with a Sciex 5500+ triple quadrupole (QQQ) mass spectrometer (AB Sciex, Singapore). The BAs were separated on a C18-PFP column (ACE, UK, 3 μm, 2.1 × 50 mm). Phase A was composed of 2 mM ammonium acetate in water, while phase B contained acetonitrile. The chromatographic gradient was configured as follows: in 0 minutes, 83% phase A and 17% phase B; in 10 minutes, 70% phase A and 30% phase B; in 13 minutes, 45% phase A and 55% phase B; and in 14 and 17 minutes, 5% phase A and 95% phase B. The last 5 minutes was used for column washing and equilibration. We used 0.4 mL min-1 as the flow rate. The BAs were ionized by a Turbo-V heated electrospray ionization source and then detected by scheduled multiple reaction monitoring modes.

### Date processing

The targeted BA annotation was based on the BA standards (25, 26). We compared the primary and secondary mass spectrometry data of the targeted BAs with the standards, as previously described. We calculated quantitative data of each sample by combining the standard curve and the area under the curve (AUC) BA values. Finally, internal standards were used for calibration. The above analyses were conducted with Analyst and OS-MQ software (AB SCIEX, Singapore).

### Statistical analysis

The BAs with missing values of more than 50% were excluded, and then we employed the K-nearest algorithm to impute the missing values (27). The molar concentration of the serum sample was calculated from the mass concentration and molecular weight. In addition, SIMCA-P software (Umetrics, Sweden) was used for orthogonal projections to latent structures discriminant analysis (OPLS-DA) (28). Benjamini-Hochberg false discovery rate (FDR) adjustment for Student’s t-test was performed on the MetaboAnalyst website (29–31). In addition, Random Forest and glmnet package were executed in R software Version 4.0.5 (R Core Team, 2021) (32). The receiver operating characteristic (ROC) curves (33), column diagrams, scatter plots and heat-maps were drawn by GraphPad Prism 9.0 (GraphPad Software Inc., USA). Besides, a biological network was generated by Cytoscape 3.8.2 (Cytoscape Consortium, USA) (34, 35).

## Results

### Study design and characteristics of BA metabolism

**Figure 1:**
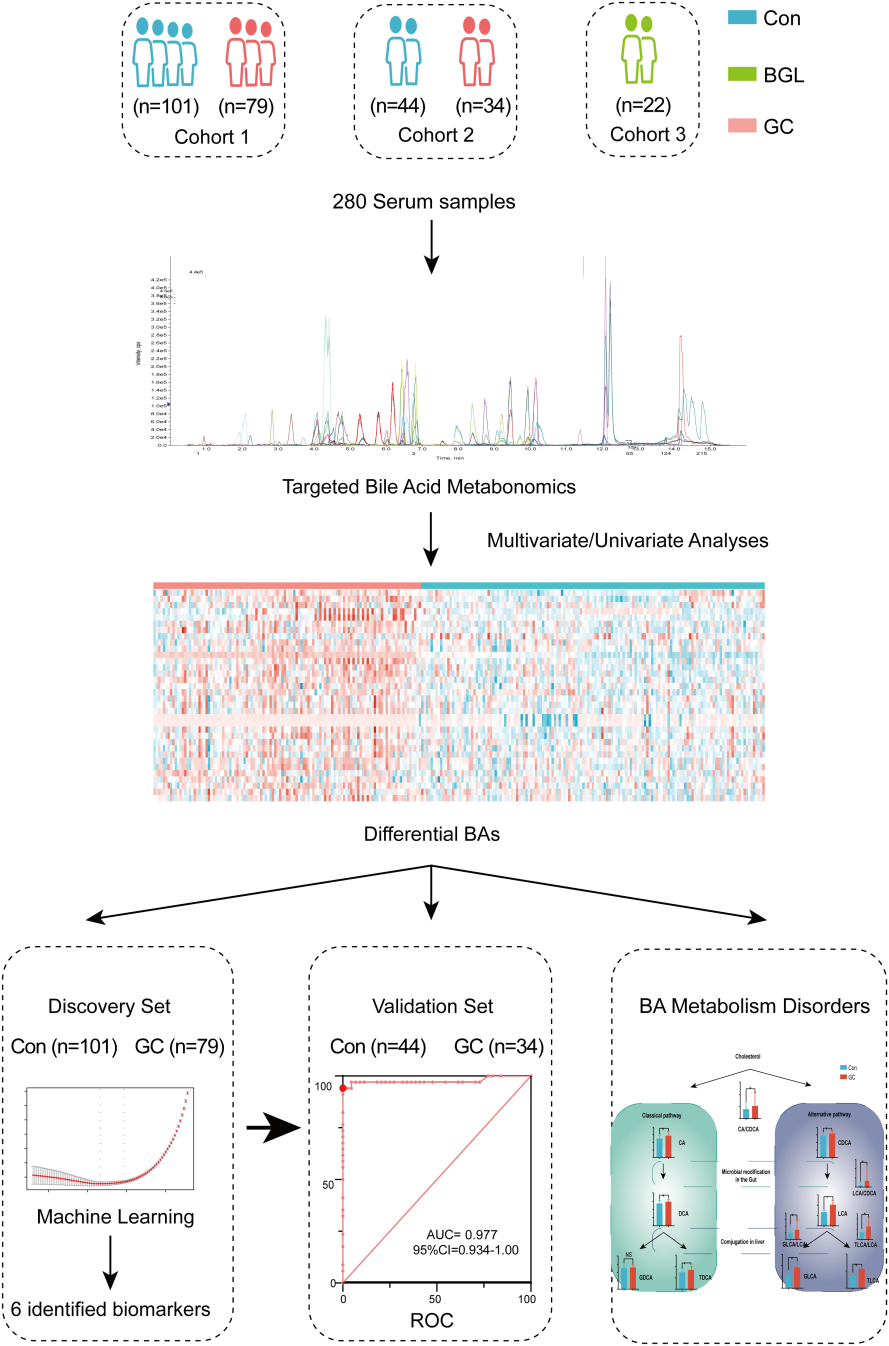
Global design and schematic representation of the study.

As shown in **Figure 1**, totally 180 participants were enrolled in the discovery cohort, including 79 GC and 101 controls. Based on the AJCC staging system, 8th edition, there were 32 patients in stage I, 9 patients in stage II, 22 patients in stage III and 16 patients in stage IV (**Figure 1**). We defined cohort 1 as the discovery set and used machine learning to conduct a diagnosis model (**Table S1**). To verify the results, cohort 2 and cohort 3 were included. Cohort 2 was validation set, including 34GC and 44Con, to detect repeatability of the model (**Table S2**). Cohort 3 included 22 BGL for verifying the differential diagnosis effect of the diagnostic model (**Table S3**). There was no significant difference in age and gender ratio (male/female) among GC and Con, whether in discovery set or validation set. Other related clinical traits were shown in **Table 1** by the form of mean ± standard deviation (SD). Besides, we presented an extracted ion chromatogram (XIC), which provided a visual representation of the analyzed targeted BAs in the GC and Con groups as shown in (**Figure S1**). Through univariate and multivariate analyses, we obtained differentially expressed BAs between the GC and Con groups. Notably, our analyses showed that there was no significant difference between the two groups in clinical features such as gender and age.

**Table 1:** Patient characteristics for the discovery and internal validation cohorts.

Characteristics	Discovery cohort (n = 180)			Validation cohort (n = 78)		
	GC (n = 79)	Con (n = 101)	p-value	GC (n = 34)	Con (n = 44)	p-value
Age (years)	63.19 ± 11.31	60.71 ± 11.92	0.21	63.65 ± 8.91	59.39 ± 12.67	0.99
Gender, male (%)	70.89	72.28	0.94	67.65	79.55	0.24
WBC (*10^9/L)	5.61 ± 1.5	6.00 ± 1.47	0.085	6.01 ± 1.69	6.11 ± 1.3	0.78
HGB (g/L)	124.14 ± 23.85	133.10 ± 41.50	0.091	121.29 ± 26.49	136.04 ± 40.97	0.074
Cre (μmol/L)	70.06 ± 17.22	70.97 ± 12.09	0.69	69.56 ± 23.79	73.52 ± 12.53	0.35
Urea (μmol/L)	12.02 ± 56.11	5.40 ± 1.14	0.25	5.65 ± 1.87	5.62 ± 1.56	0.94
Glucose (mmol/L)	5.49 ± 2.01	5.58 ± 1.41	0.72	5.24 ± 1.14	7.36 ± 13.11	0.35
ALT (U/L)	15.65 ± 10.4	23.24 ± 13.90	<0.001	14.91 ± 9.04	22.07 ± 12.97	0.0078
AST (U/L)	18.37 ± 7.63	21.94 ± 6.32	<0.001	17.12 ± 5.61	21.07 ± 6.06	0.0044
TBIL(μmol/L)	12.82 ± 5.58	16.02 ± 5.92	<0.001	11.33 ± 4.37	15.16 ± 6.34	0.004
DBIL(μmol/L)	3.6 ± 2.09	5.16 ± 5.03	0.012	3.48 ± 1.65	4.63 ± 2.07	0.011
TBA (μmol/L)	5.93 ± 5.83	—	—	4.52 ± 2.43	—	—
Cancer Stage						
I	32	—	—	15	—	—
II	9	—	—	1	—	—
III	22	—	—	12	—	—
IV	16	—	—	6	—	—
CEA (ng/ml)	8.19 ± 18.69	—	—	7.02 ± 22.75	—	—
CA199 (U/mL)	54.25 ± 170.26	—	—	154.19 ± 492.32	—	—

Data are presented as mean ± SD. WBC, white blood cell; HGB, hemoglobin; Cre, creatinine; ALT, alanine transaminase; AST, glutamic oxaloacetic transaminase; TBIL, total bilirubin; DBIL, direct bilirubin; TBA, total bile acid; CEA, carcinoembryonic antigen; CA199, carbohydrate antigen 199.

**Figure 2:**
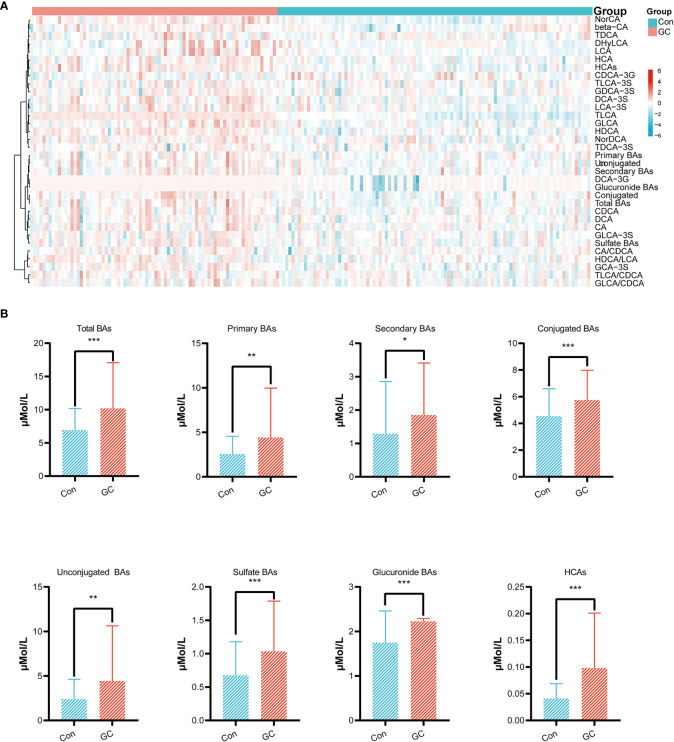
Overview of Bile acids. Differential expression of BAs, BA ratios and total concentration of BA sub-classes between the GC and Con groups were plotted as heat-map **(A)**. In this plot, red represents a higher concentration, while blue represents a lower concentration. Concentrations of eight kinds of BA sub-classes in the GC and Con group. For all figures, FDR-adjusted Q-value: *Q < 0.05; **Q < 0.01; ***Q < 0.001 **(B)**.

After screening out the BAs with excess missing values or unstable detection, we obtained a total of 49 BAs. We then added biologically significant ratios and total concentrations of sub-classes to then BAs. By calculating the BAs (ratios or sub-classes) with significant differences between the GC and Con groups, a heat-map of 34 features was drawn (**Figure 2A**). Overall, patients in the GC group had higher total BA concentration compared with the Con group. Interestingly, in the GC group, the levels of some BA sub-classes of interest, including conjugated BAs, unconjugated BAs, sulfate BAs, glucuronide BAs and HCAs showed different degrees of increase (**Figure 2B**).

### Correlation analysis of the BAs and clinical traits

**Figure 3:**
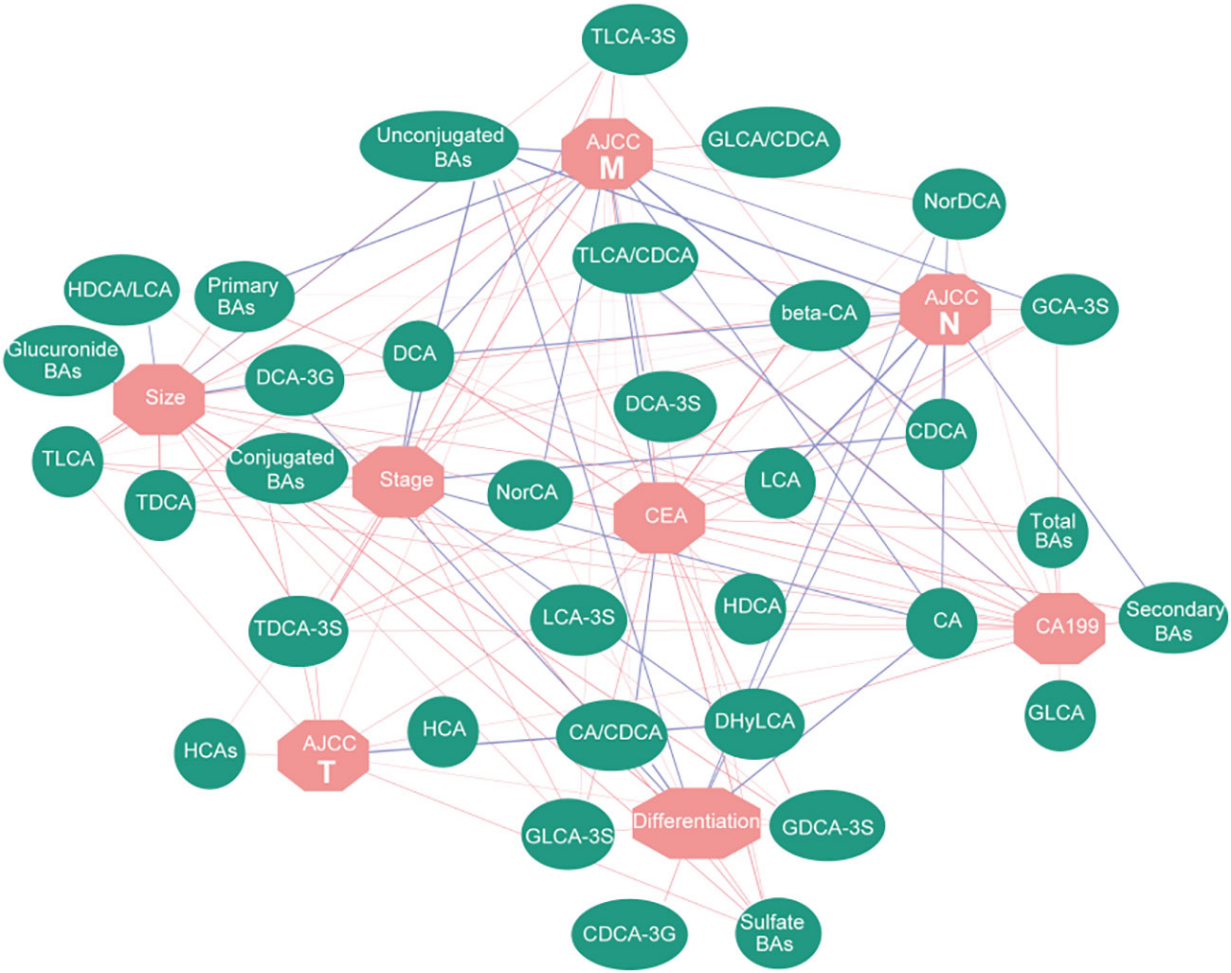
Correlation network based on Spearman correlation analysis. It shows the correlation between BAs and clinical traits. Red lines represent positive correlation, while blue lines represent negative correlation; the width of the line represents the correlation coefficient.

To analyze the correlation among the serum BAs and clinical traits in the patients with GC, we performed a Spearman correlation analysis. We selected eight clinical traits related to GC, including Size, CEA or Stage, which represented the pathological features of GC. We separately classified the BAs strongly correlated with each clinical trait (**Figure 3**; **Table S6**). These data indicated that the BAs have commendable reactivity with clinical traits related to GC and could be characteristic biomarkers for clinical diagnosis of GC.

### Identification of diagnostic markers for GC

Due to lack of effective screening tools and diagnostic markers for GC, we used targeted quantitated BAs to construct a diagnostic signature. LASSO regression analysis based on glmnet R package was performed to screen BA biomarkers that could be used for GC diagnosis. Six BAs (ratio), including HCA, TLCA, NorCA, DCA-3G, TLCA-3S and HDCA/LCA were obtained (**Table 2**).

**Table 2:** Characteristics of differential expression of the six markers for GC detection identified in this study.

Biomarker	P-value	Q-value	Log2 (Fold Change)	AUC of ROC
HCA	9.38E-05	0.00048	0.81	0.65 (95% CI:0.58-0.71)
TLCA	4.64E-67	3.57E-65	1.32	0.99 (95% CI:0.97-1)
NorCA	1.34E-13	5.16E-12	0.81	0.79(95% CI:0.73-0.84)
DCA-3G	7.17E-12	1.84E-10	0.38	0.70 (95% CI:0.64-0.77)
TLCA-3S	0.0016	0.0056	0.67	0.60 (95% CI:0.53-0.67)
HDCA/LCA	0.0061	0.016	0.91	0.62 (95% CI:0.55-0.69)

**Figure 4:**
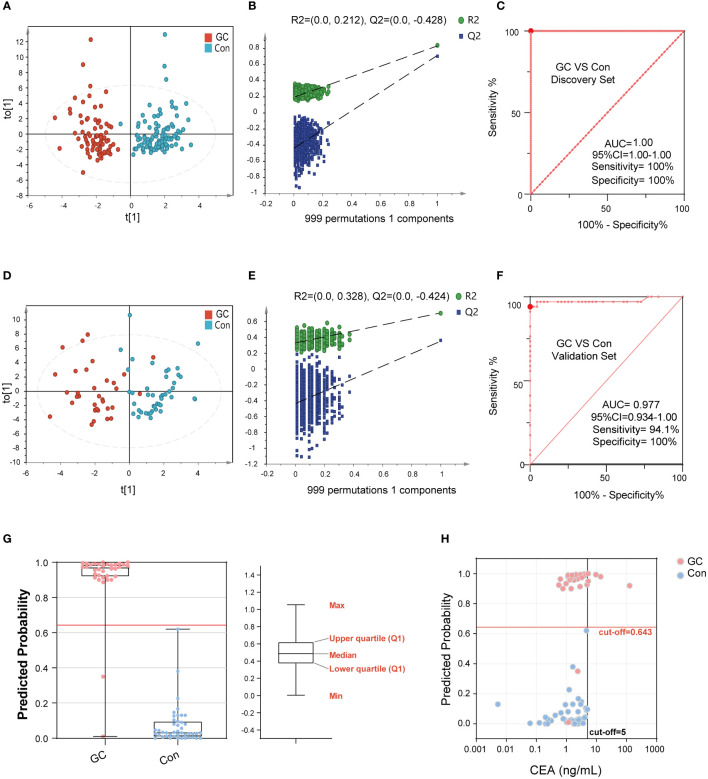
The performance of BA biomarkers in distinguishing GC and Con group. The OPLS-DA score plot of the GC and Con groups in discovery set **(A)**. The permutation test result of the above OPLS-DA, which could verify whether the classification is overfitted **(B)**. The ROC curve of 6-BAs diagnostic panel between the GC and Con group, which was constructed using the discovery set **(C)**. The OPLS-DA score plot of the GC and Con groups in the validation set **(D)**. The permutation test result of the above OPLS-DA. **(E)** The ROC curve of 6-BAs diagnostic panel between the GC and Con group, which was constructed in discovery set and test in validation set **(F)**. Predicted probability of the 6-BAs diagnostic panel identified in the discovery set and applied to the validation set **(G)**. Scatter plot for comparing the 6-BAs diagnostic panel and CEA. The CEA concentration of the Con is randomly selected from the upper limit of the normal value and zero **(H)**.

The discovery set was used to construct the diagnostic panel. The OPLS-DA score plot showed an obvious separation between the GC group and the Con group (**Figure 4A**). To validate the classification, we performed a permutation test (**Figure 4B**). The results showed that the Y-axis intercept of R2 and Q2 was 0.212 and -0.428 (usually R2 and Q2 were less than 0.4 and 0, respectively), with a significant positive slope, which indicated that the data in the discovery set was not overfitted and reliable. Surprisingly, in the discovery set, the predictive ability of the GC diagnostic panel based on the RF model was perfect, and its sensitivity and specificity were 100%. Meanwhile, the AUC value of the model was 1 (95%CI: 1.00-1.00) (**Figure 4C**). Thereafter, we included another cohort as a validation set to evaluate the diagnostic model. As shown in **Figure 4D**, there were significant differences between the GC and the Con groups. Similarly, the verification data was not overfitted (**Figure 4E**). In the validation set, the predictive ability of the GC diagnostic panel was also satisfactory. Its sensitivity and specificity were 94.1% and 100%, respectively (**Figure 4F**). In addition, the AUC value of the verification set model was 0.98 (95%CI: 0.93-1.00). Besides, among all the GC and Con samples in the combined set, age (60 years old as the boundary) and gender had no significant effect on the predicted ability of the diagnostic panel (**Figure S2**). In addition, there was no correlation between the predicted ability of the diagnostic panel and whether GC was in an advanced stage, nor with AJCC tumor stage (**Figure S3**). These data showed that the diagnostic ability of the developed diagnostic panel was free from tumor load, which makes it an optimal diagnostic tool for the detection of GC.

The calculated model cut-off value in the discovery set was applied to the validation set as shown in **Figure 4G**. Participants with a prediction probability of more than 0.64 were categorized as GC; otherwise, they were classified as Con group. To visually demonstrate the difference between the diagnostic panel and the CEA, a scatter plot was created to distinguish GC from Con (**Figure 4H**). It was not surprising to observe that CEA had superior sensitivity in distinguishing GC from Con, but lacked specificity. Together, the BA diagnostic panel showed promising sensitivity and specificity in the diagnosis of GC.

### Identification of diagnostic markers for EGC

**Figure 5:**
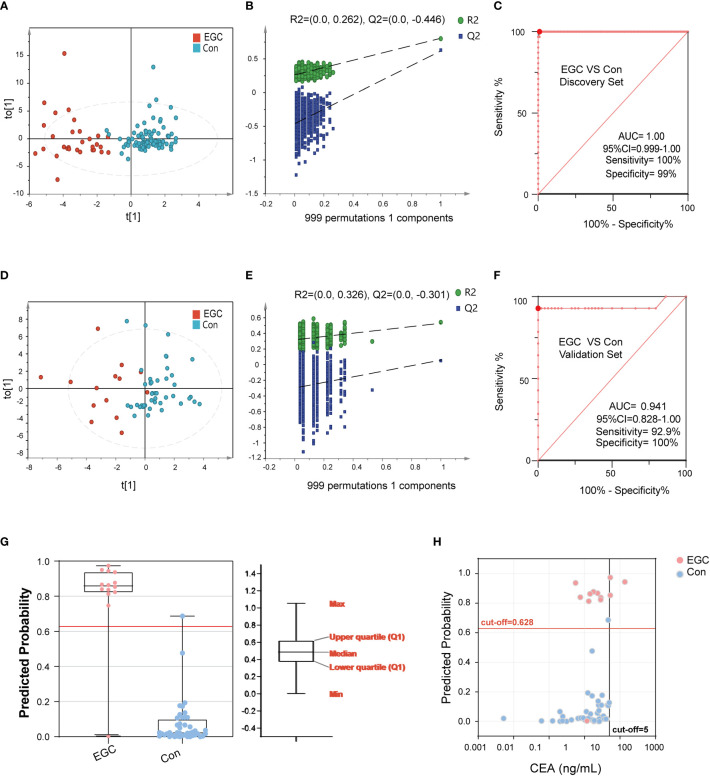
The performance of BA biomarkers in distinguishing early GC and Con group. **(A)**: OPLS-DA score plot of early GC and Con groups in the discovery set. **(B)**: permutation test result of the above OPLS-DA. **(C)**: ROC curve of 6-BAs diagnostic panel between early GC and Con group, which was constructed in the discovery set. **(D)**: OPLS-DA score plot of early GC and Con groups in the validation set. **(E)**: permutation test result of the above OPLS-DA. **(F)**: ROC curve of 6-BAs diagnostic panel between early GC and Con group, which was constructed in the discovery set and validation set. **(G)**: Predicted probability of the 6-BAs diagnostic panel identified in the discovery set and applied to the validation set. **(H)**: Scatter plot for comparing the 6-BAs diagnostic panel and CEA.

Early diagnosis is crucial for the prognosis of GC. Here, we constructed a diagnostic model for EGC. Our analysis showed that there are obvious metabolic differences between the EGC group and the Con group (**Figure 5A**). Likewise, the data of the discovery set was not overfitted (**Figure 5B**). The Y-axis intercept of R2 and Q2 was 0.262 and -0.446, respectively, with a positive slope. Similarly, the ROC curve was also surprisingly perfect, and its sensitivity and specificity were 100% and 99%, respectively (**Figure 5C**). Besides, the AUC value of this model was 1 (95%CI: 0.999-1.00). In addition, the diagnostic model of the verification set yielded better results, and the OPLS-DA clearly separated the EGC group from the Con group (**Figure 5D**). Moreover, the data from the verification set was still not overfitted (**Figures 5E**, **S4A**). Unexpectedly, as shown in **Figure 5F**, the prediction rate of ROC curve remained high (Sensitivity=92.9%, Specificity=100%, 95%CI: 0.83-1.00, AUC=0.94). As previously mentioned, we used the same method to determine a cut-off value of 0.63, which distinguished the EGC from the Con (**Figures 5G, H**).

### Identification of differential diagnostic markers for GC and BGL

**Figure 6:**
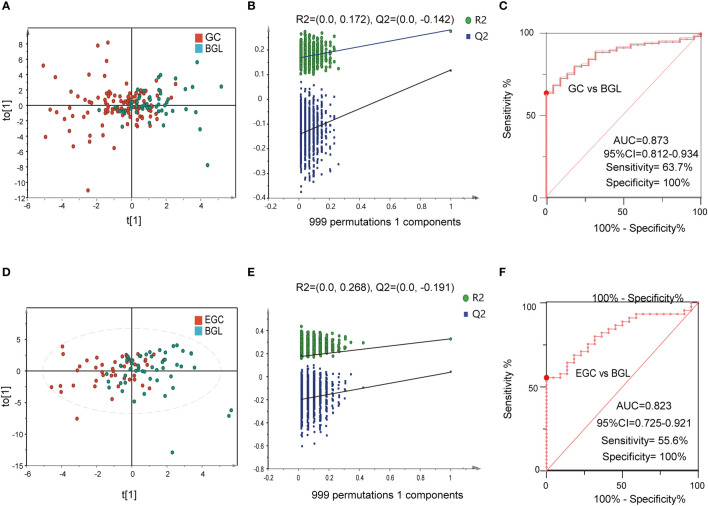
The performance of BA biomarkers in distinguishing GC and BGL group. **(A)**: OPLS-DA score plot of GC and BGL groups. **(B)**: permutation test result of the OPLS-DA result in **(A, C)**: ROC curve of 6-BAs diagnostic panel between GC and BGL group. **(D)**: OPLS-DA score plot of early GC (EGC) and BGL groups. **(E)**: permutation test result of the OPLS-DA result in **(D, F)**: ROC curve of 6-BAs diagnostic panel between EGC and BGL group.

The differential diagnosis between BGL and GC can help doctors to make a preliminary judgment, whether the disease is benign or malignant, without relying on pathological examination. Hence, we used 22 cases of BGL and 113 cases of GC to further verify the differential diagnostic efficacy of 6-BAs diagnostic panel. The results suggested that there were significant metabolic differences between GC and BGL group (**Figure 6A**). Meanwhile, this model did not perform a tendency of overfit (**Figure 6B**). The AUC value of ROC plot was 0.873 (95%CI: 0.812-0.934), which sensitivity and specificity were 63.7% and 100% respectively (**Figure 6C**). Likewise, The OPLS-DA score plot of EGC and BGL group also suggested that there were metabolic differences between BGL and the early stage of gastric cancer (**Figure 6D**). This model also has not been overfitted (**Figures 6E**, **S4B**). Finally, we found that for EGC, the AUC value of the ROC curve was 0.823 (95%CI: 0.725-0.921). Its sensitivity decreased to 55.6%. However, the specificity remains 100%. Obviously, unlike disease screening, differential diagnosis paid more attention to specificity. Therefore, 6-BAs diagnostic panel can also be used as a marker for differential diagnosis.

## Discussion

In this study, we profiled serum BAs of 280 GC patients, BGL patients and healthy controls using targeted BA metabolomics. Our data showed that the total serum BA pool was significantly altered in the patients with GC. Moreover, these differential BAs were associated with clinical traits. Besides, we constructed a diagnostic panel using a machine learning algorithm consisting of six BA molecules. The constructed model demonstrated good diagnostic efficiency for BGL and EGC. The metabolism of BAs can be considered as a crucial indicator to the prevention and treatment of GC.

**Figure 7:**
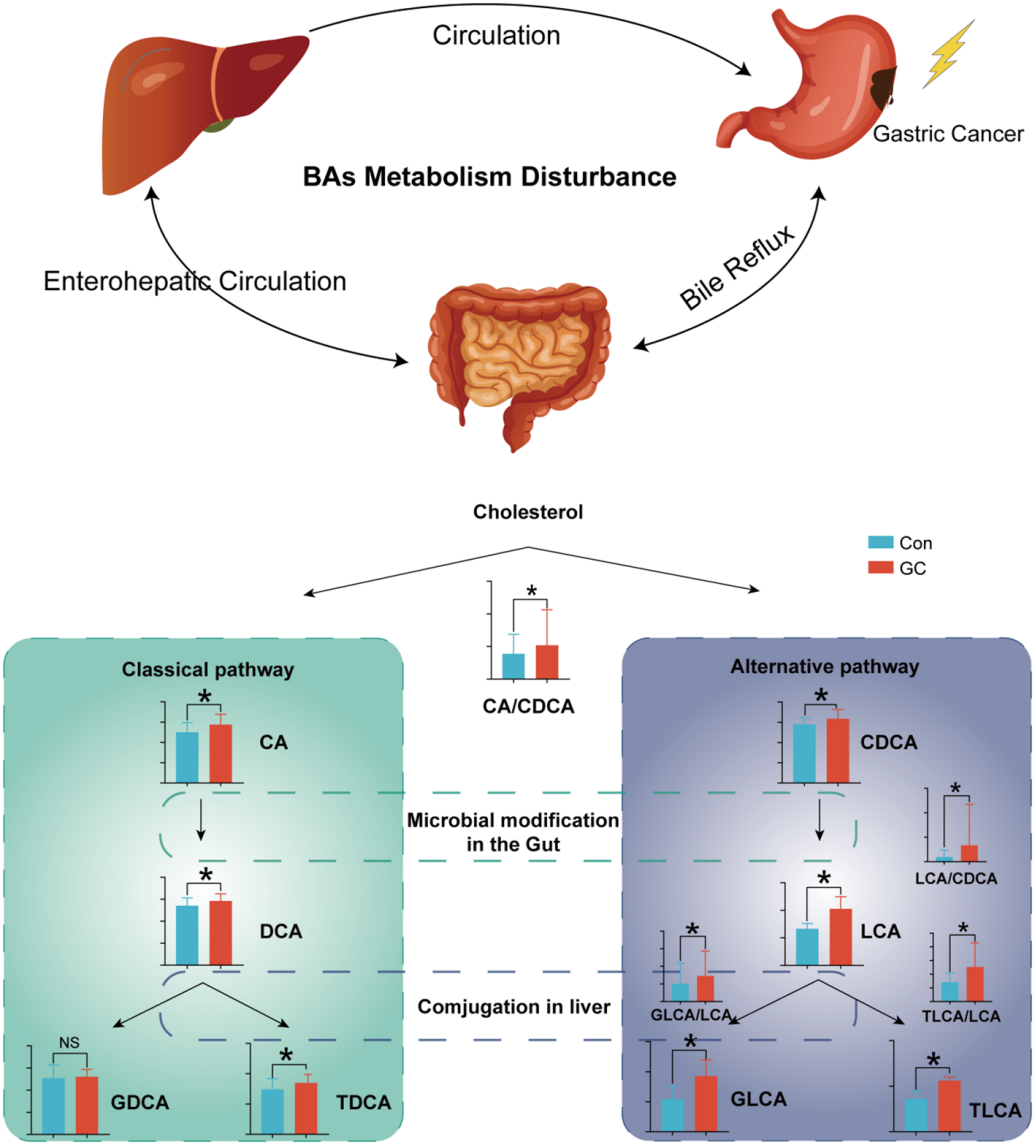
Overview of the BA metabolic dysregulation in GC. FDR-adjusted Q-value: *Q < 0.05.

Recent studies have shown that BAs are important signal molecules. In biological systems, BAs maintain glucose and lipid metabolism homeostasis and energy expenditure by acting on the receptors in peripheral tissues and organs, such as Farnesol X receptor FXR and TGR-5 (36–38). BAs are mainly metabolized through enterohepatic circulation and play a crucial role in regulating the functions of digestive tract and intestinal immunity (39). Besides, studies have shown that BAs are important regulatory molecules in tumors. In HCC, BAs were shown to directly incapacitate the plasma membrane, leading to the activation of protein kinase C (PKC), which activated the P38-MAPK pathway, resulting in increased activation of p53 and nuclear factor κB (NF-κB) which mediate cellular apoptosis and inflammation (40). In this study, there were significant metabolic changes in BAs in patients with GC. Increased synthesis of hepatic BAs or reabsorption of intestinal BAs led to an increased pool volume of the BAs. Improved BAs pool in GC may be beneficial in disease development. Besides, glucuronidated BAs and sulfated BAs were significantly increased in GC (**Figure 7**). The pathophysiological role of the BAs in GC remains unclear.

Pathological characteristics such as tumor stage and grade accurately reflects the degree of malignancy of cancers, and these characteristics have a strong guiding significance for clinic (41). However, these features can often only be accurately captured in an invasive fashion. Markers which are easily obtained under a noninvasive manner reflecting disease characteristics are urgently needed. Correlation analysis showed that BAs were closely related to T stage, N stage and grade, etc. Circulating BAs can be used as indicators reflecting the characteristics of GC.

The ratio of upstream and downstream molecules in the metabolic pathway can indirectly reveal the change of corresponding catalytic enzyme activity. By comparing the ratio of expression of adjacent metabolites, we demonstrated that there was enhancement of the metabolic activity of both the classical and alternative pathways in GC (42). Among these, the classical pathway has been more emphasized (CA/CDCA). However, there is a dramatic occurrence of metabolic disorders in the alternative pathway. Increased LCA/CDCA in GC indicated that intestinal flora enhances the catalytic activity of primary BAs CDCA, resulting in increased cytotoxic LCA. With the increased LCA uptake in the intestinal tract, there was increased activity of LCA-modifying enzymes in the liver (GLCA/LCA, TLCA/LCA). CDCA, LCA, TLCA are hydrophobic and cytotoxic, which lead to cell damage and inflammation (43, 44).

In this study, the diagnostic model constructed by the 6 molecules and ratios in GC had good diagnostic performance, even in EGC. The constructed panel had a high sensitivity and specificity for screening high-risk GC populations. Moreover, the diagnostic efficiency of the diagnostic model was independent of tumor load, which is an important biomarker for early cancer screening. Screening for early-stage cancer is the most critical step in the improvement of the current GC status, as these patients undergo less trauma, fewer complications and better prognosis. Among the 6 molecules, TLCA expression was the most differentially expressed metabolite, with an AUC of 0.99 (95% CI:0.97-1). TLCA was the strongest activator of TGR5 among all the BAs and promoted the occurrence of liver cancer by activating the TGR5 (45). Remodeling of energy metabolism is a common feature in tumor metabolic reprogramming. Hyocholic acid species (HCA and HDCA/LCA) play an important role in regulating insulin sensitivity, glucose homeostasis and energy expenditure (46).

The metabolism of BAs is mainly influenced by the catalysis of liver and intestinal microbiota. However, different diseases have been found to have specific features of BAs metabolism (42, 47). The diagnostic specificity can be enhanced by using multiple molecular model. Compared with a single molecule, our model is composed of six molecules and ratios which can reduce molecular noise and increase the accuracy of diagnosis. These 6 BAs could make up for the lack of non-invasive markers for GC screening and resolve the cost associated with endoscopy as a screening tool for GC.

We used an in-depth metabolomics approach which provided high-quality and information-rich BAs data in patients with GC. Differential BAs, including many low-abundant BAs, were annotated based on the BA standards. Due to limited detection methods, data on the low-abundance BAs remain scanty. Recent data has shown that some of these BAs are closely related to human health. Besides, BAs play a key role in regulating gastric mucosal homeostasis, which mediates stomach upset. Thus, the newly reported BAs could serve as screening markers. In addition, these molecules provide a new tool in understanding the pathophysiology of GC.

Although our study had an important outcome, there is a need to confirm the diagnostic value of those candidates in a multi-center large sample cohort. In addition, the molecular mechanism of BA metabolism disorder on the occurrence and development of GC needs further evaluation in cell assays.

## Conclusion

Taken together, our data demonstrated that the BAs metabolism disorder is involved in GC development. Our diagnostic model using 6 BAs or ratio provided promising diagnostic efficiency for GC, which could perform early screening of high-risk populations and promote early diagnosis of GC.

## Data availability statement

The original contributions presented in the study are included in the article/**Supplementary Materials**. Further inquiries can be directed to the corresponding authors.

## Ethics statement

The studies involving human participants were reviewed and approved by the Ethics Committee of First Affiliated Hospital of Dalian Medical University. The patients/participants provided their written informed consent to participate in this study.

## Author contributions

CP: data curation, formal analysis, software, methodology, writing - original draft, and writing - review and editing. DD: data curation, formal analysis, investigation, methodology, project administration, writing - original draft, and writing - review and editing. TW: data curation, formal analysis, investigation, and methodology. ZW: methodology, formal analysis, resources, and validation. BZ: data curation and formal analysis. QY: data curation and formal analysis. GL: conceptualization, resources, validation, and supervision. YL: conceptualization, resources, project administration, validation, and supervision. PY: conceptualization, resources, validation, supervision, funding acquisition, project administration, writing - review and editing. All authors contributed to the article and approved the submitted version.

## Funding

This research was funded by The Key Research And Development Project Of Liaoning Province (No. 2018225054) and The National Natural Science Foundation of China (No. 81873156).

## Conflict of interest

ZW and PY are co-founders of iPhenome Yun Pu Kang Biotechnology Inc. ZW is an employee of iPhenome Yun Pu Kang Biotechnology Inc.

The remaining authors declare that the research was conducted in the absence of any commercial or financial relationships that could be construed as a potential conflict of interest.

## Publisher’s note

All claims expressed in this article are solely those of the authors and do not necessarily represent those of their affiliated organizations, or those of the publisher, the editors and the reviewers. Any product that may be evaluated in this article, or claim that may be made by its manufacturer, is not guaranteed or endorsed by the publisher.

## Abbreviations

AD, Alzheimer’s disease; AUC, area under the curve; BA, bile acid; CEA, carcinoembryonic antigen; CI, credible intervals; DCA, deoxycholic acid; DCA-3G, deoxycholic acid 3-glucuronide; ECG, early gastric cancer; FDR, false discovery rate; GC, gastric cancer; HCA, hyocholic acid; HCC, hepatocellular carcinoma; HDCA, hyodeoxycholic acid; IHC, immunohistochemistry; LCA, lithocholic acid; NF-κB, nuclear factor κB; NorCA, norcholic acid; OPLS-DA, orthogonal projections to latent structures discriminant analysis; PKC, protein kinase C; QC, quality control; ROC, receiver operating characteristic; SD, standard deviation; TLCA, taurolithocholic acid; TLCA-3S, taurolithocholic acid 3-sulfate; XIC, extracted ion chromatogram.
