# Supplementary material for: Metabolomics study identified bile acids as potential biomarkers for gastric cancer: A case control study
Source: Front Endocrinol (Lausanne). 2022 Nov 18;13:1039786. doi: 10.3389/fendo.2022.1039786 (PMC9715751; doi:10.3389/fendo.2022.1039786)
Supplement: Supplementary file 1 [file DataSheet_1.pdf]

A

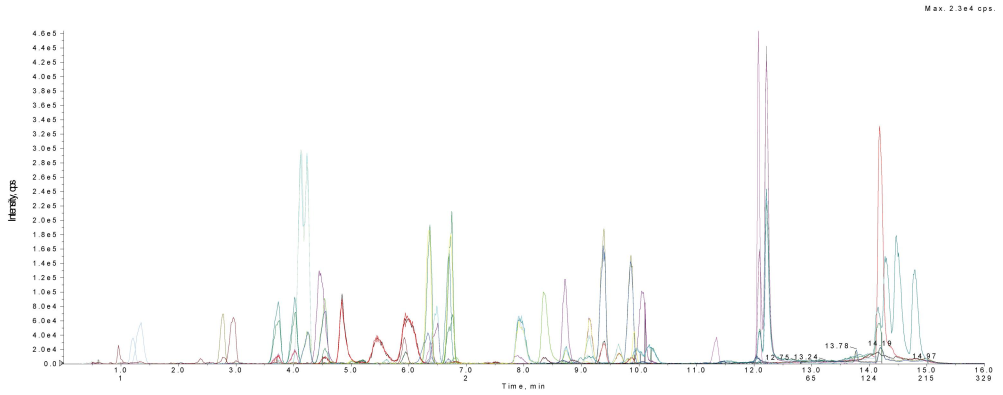

B

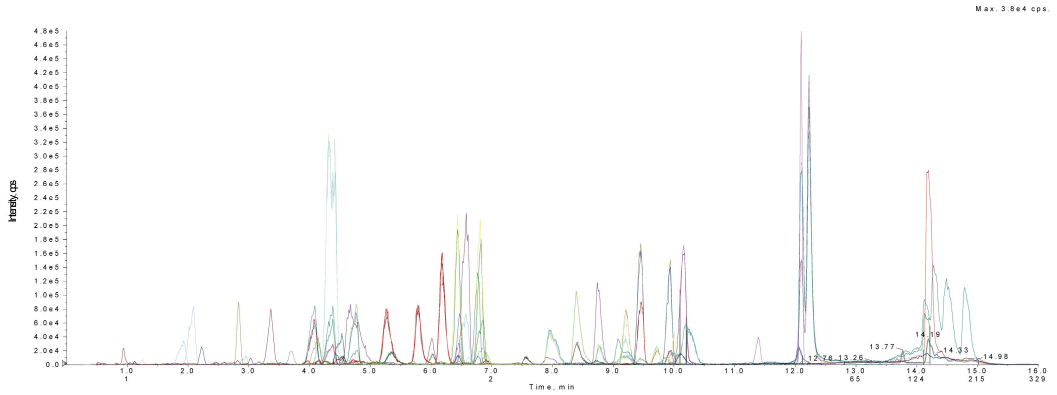

Figure S1 The representative extracted ion chromatograms (XIC) in the GC (A) and Con (B) group.

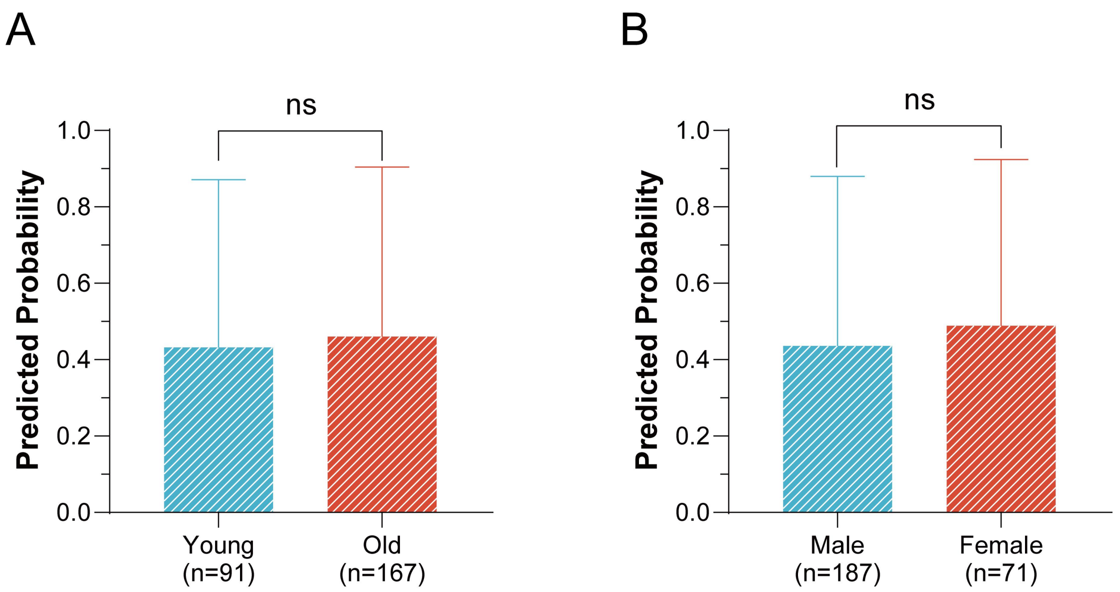

Figure S2 In the combined set (discovery set and validation set), samples were regrouped based on age (A) and gender (B). These two plots show the predicted probability in each group. For all figures, FDR-adjusted Q-value: ns means not significant (Q > 0.05).

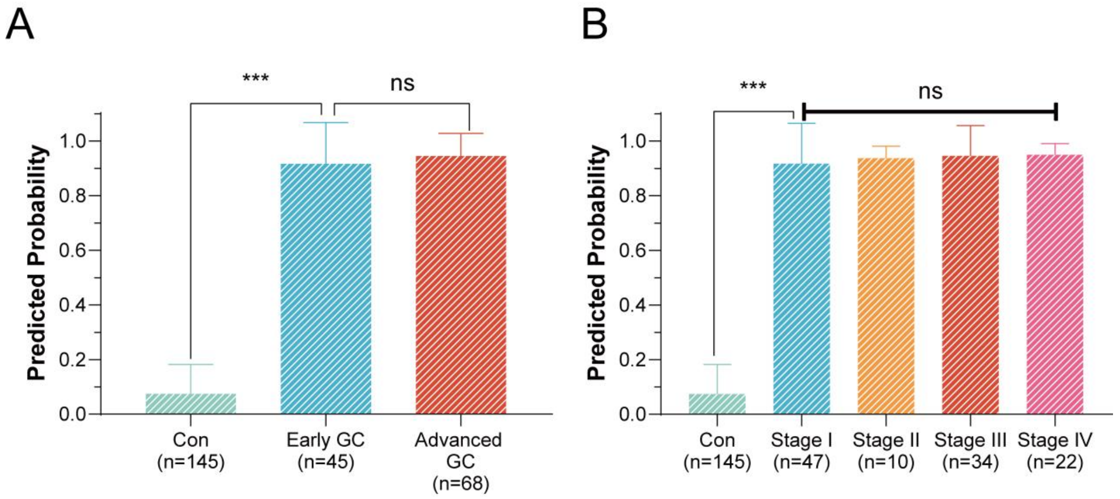

Figure S3 In the combined set (discovery set and validation set), samples were regrouped based on early/advanced stage (A) and AJCC stage (B).These two plots show the predicted probability in each group. For all figures, FDR-adjusted Q-value: ns, not significant (Q > 0.05); ***Q < 0.001.

A

The OOB error is 0.069			
	Con	EGC	class.error
Con	42	2	0.0455
EGC	2	12	0.143

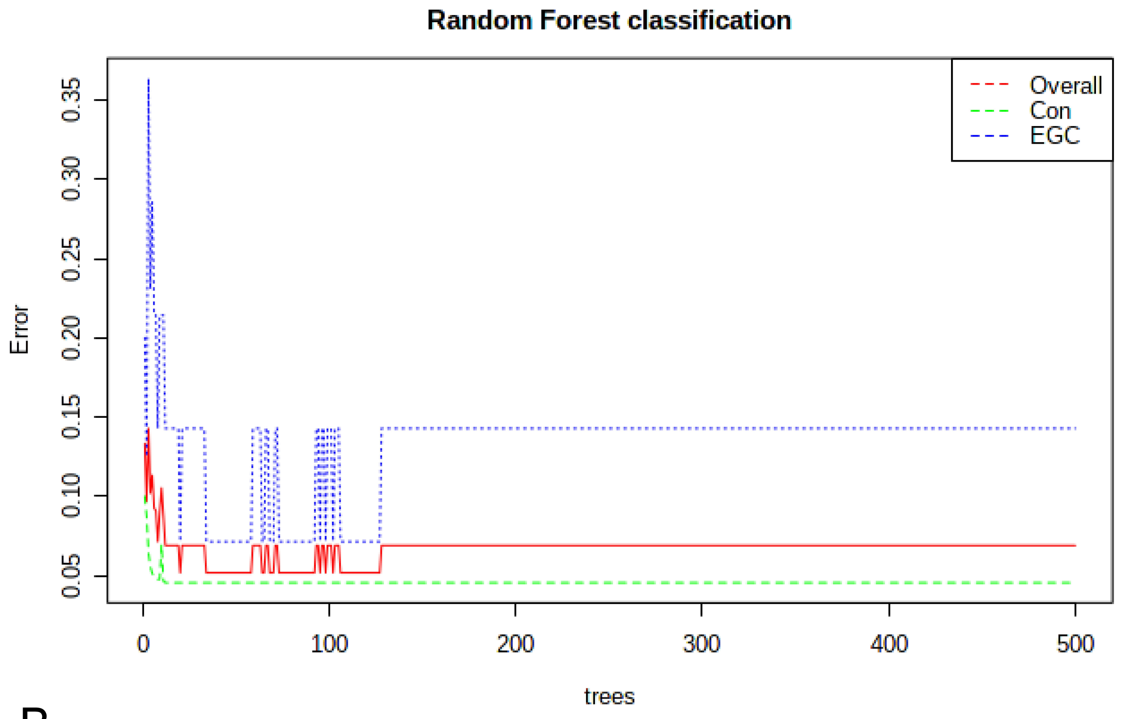

B

The OOB error is 0.269			
	BGL	EGC	class.error
BGL	11	11	0.5
EGC	7	38	0.156

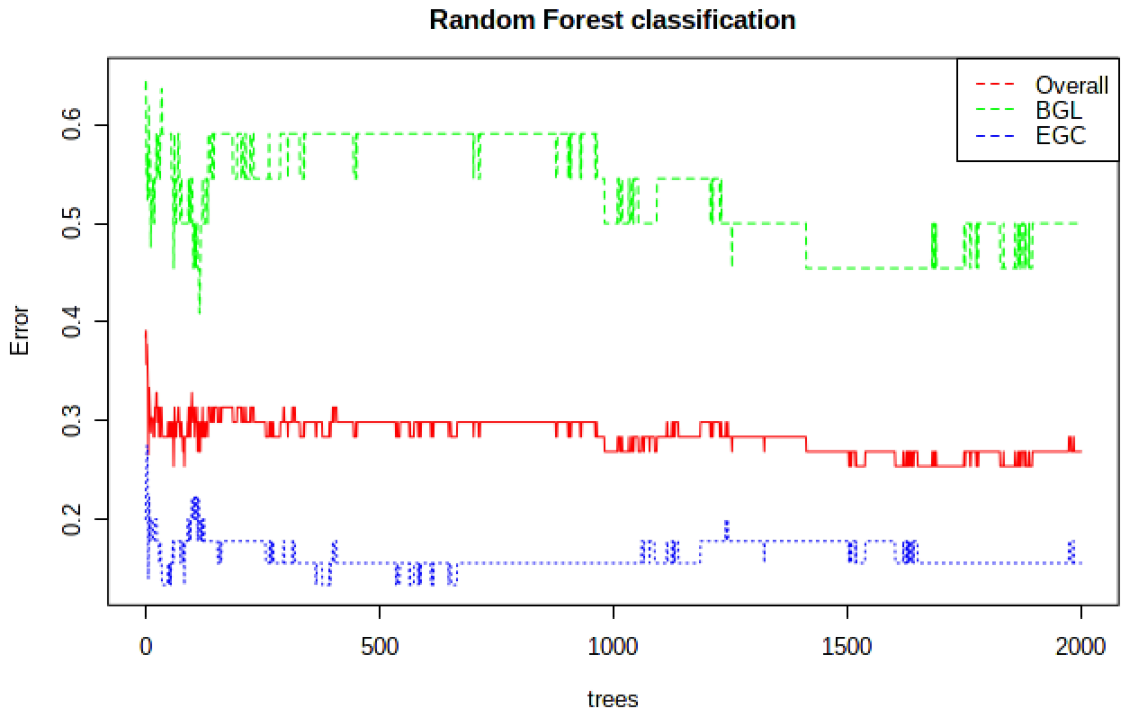

Figure S4 The random forest model robustness assessment in EGC-Con (A) and EGC-BGL (B)
